# A99 INCREASING THE NUMBER OF PASSES FOR ENDOSCOPIC ULTRASOUND-GUIDED FINE NEEDLE ASPIRATION BIOPSY OF SOLID MASS LESIONS – A QUALITY IMPROVEMENT INITIATIVE TO IMPROVE DIAGNOSTIC YIELD

**DOI:** 10.1093/jcag/gwae059.099

**Published:** 2025-02-10

**Authors:** G Sandha, S Khan, P Mathura, L Puttagunta, S Girgis, A Thiesen, J Zhang, J Nilsson, S Wasilenko, S Zepeda-Gomez

**Affiliations:** University of Alberta College of Health Sciences, Edmonton, AB, Canada; University of Alberta College of Health Sciences, Edmonton, AB, Canada; University of Alberta College of Health Sciences, Edmonton, AB, Canada; University of Alberta College of Health Sciences, Edmonton, AB, Canada; University of Alberta College of Health Sciences, Edmonton, AB, Canada; University of Alberta College of Health Sciences, Edmonton, AB, Canada; Alberta Health Services, Edmonton, AB, Canada; University of Alberta College of Health Sciences, Edmonton, AB, Canada; University of Alberta College of Health Sciences, Edmonton, AB, Canada; University of Alberta College of Health Sciences, Edmonton, AB, Canada

## Abstract

**Background:**

A retrospective chart audit (01/2022-12/2022) of endoscopic ultrasound (EUS)-guided fine needle aspiration biopsy (FNAB) of solid mass lesions revealed a disappointing diagnostic yield of 56% with a single needle pass. To improve this, a quality improvement (QI) intervention where endoscopists (three) were encouraged to perform three needle passes per patient was developed and trialed.

**Aims:**

To assess intervention impact on improving the diagnostic yield of EUS-FNAB of solid mass lesions over a 9-month study period.

**Methods:**

A chart audit was completed quarterly for all patients undergoing EUS-FNAB of solid mass lesions from 01/2024-09/2024. Descriptive statistics were completed. Only a definite diagnosis, as confirmed on histological examination, was considered when calculating the diagnostic yield.

**Results:**

A total of 183 patients (112 M, 71 F), mean age 63±13 years (range 12-88 years), underwent 198 EUS-FNABs by 3 endoscopists over 9 months. A single pass with an FNAB needle was undertaken in 36/198 cases (18%). The diagnostic yield was 20/36 (56%), similar to the pre-intervention year. The solid mass lesions targeted were pancreas (16), lymph nodes (12), subepithelial (3), rectal and retro-peritoneal (2 each), and ampulla (1). One endoscopist performed 25/36 cases (69%) whereas the other two performed 11/36 (31%) and 0/36 cases. Proximity to vasculature and technical difficulty were reasons provided in 11 and 1 case(s), respectively, whereas no reason was documented in 24 cases. There was no difference in these variables between the groups with a definite diagnosis (20/36) vs. those without (16/36). After the first quarter audit, the need to avoid a single needle-pass was reinforced. Figure 1 shows the trend of single vs. 3 or more needle passes for the pre-intervention vs. the post-intervention year. There is a trend towards performing fewer single needle passes (39 pre vs. 36 post) and increasing 3 or more needle passes (25 pre vs. 68 post). This was associated with an improvement in diagnostic yield in the pre (22/39 [56%] vs. 21/25 [84%]) vs. post groups (20/36 [56%] vs. 61/68 [90%]).

**Conclusions:**

Education, regular audit, and continued reinforcement has demonstrated an improvement in the diagnostic yield of EUS-FNAB by increasing the number of needle passes to 3 or more for solid mass lesions.

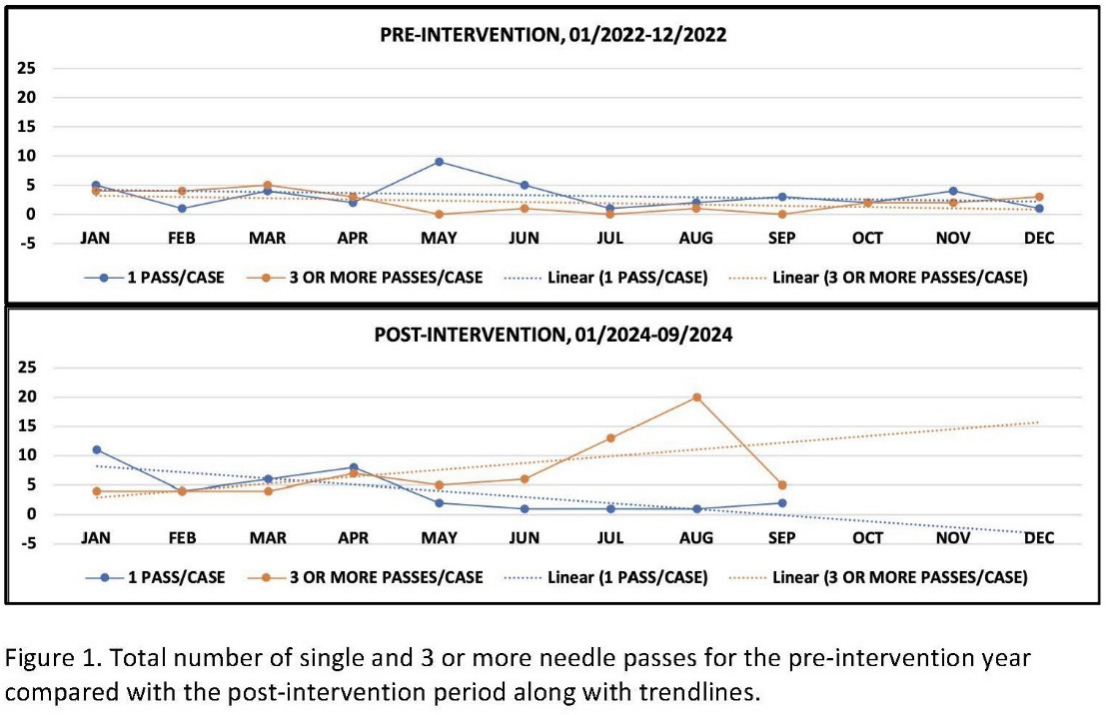

**Funding Agencies:**

None

